# IRF6 polymorphisms in Brazilian patients with non-syndromic cleft lip with or without palate^☆^

**DOI:** 10.1016/j.bjorl.2019.04.011

**Published:** 2019-06-08

**Authors:** João Felipe Bezerra, Heglayne Pereira Vital da Silva, Raul Hernandes Bortolin, André Ducati Luchessi, Marcela Abbott Galvão Ururahy, Melina Bezerra Loureiro, Vera Lúcia Gil-da-Silva-Lopes, Maria das Graças Almeida, Viviane Souza do Amaral, Adriana Augusto de Rezende

**Affiliations:** a Universidade Federal do Rio Grande do Norte, Departamento de Análises Clínicas e Toxicológicas, Natal, RN, Brazil; b Universidade de São Paulo (USP), Departamento de Análises Clínicas e Toxicológicas, São Paulo, SP, Brazil; c Universidade Estadual de Campinas, Escola de Ciências Médicas, Departamento de Genética Médica, Campinas, Campinas, SP, Brazil; d Universidade Federal do Rio Grande do Norte, Departamento de Biologia Celular e Genética, Natal, RN, Brazil

**Keywords:** Nonsyndromic orofacial clefts, Etiology, IRF6, Single nucleotide polymorphism, Fendas orofaciais não sindrômicas, Etiologia, IRF6, Polimorfismo de um único nucleotídeo

## Abstract

**Introduction:**

Non-syndromic orofacial clefts have a complex etiology due to the contribution from both genetic and environmental risk factors, as well as the interaction between them. Among the more than 15 susceptibility loci for non-syndromic orofacial clefts with considerable statistical and biological support, the IRF6 is the most validated gene by the majority of studies. Nonetheless, in genetically heterogeneous populations such as Brazilian, the confirmation of association between non-syndromic orofacial clefts and IRF6 common variants is not a consolidated fact and unrecognized IRF6 variants are poorly investigated.

**Objective:**

The aim of this study was to investigate the association of IRF6 polymorphisms with non-syndromic orofacial clefts development in a population from northeast Brazil.

**Methods:**

Blood samples of 186 non-syndromic orofacial clefts patients and 182 controls from Rio Grande do Norte, Brazil, were obtained to analyze IRF6 polymorphisms (rs2235371, rs642961, rs2236907, rs861019, and rs1044516) by real-time polymerase chain reaction. Non-syndromic orofacial clefts patients were classified in cleft lip and palate, cleft palate only and cleft lip only groups.

**Results:**

The genotype and allele frequencies of single nucleotide polymorphism rs2235371 in IRF6 showed significant differences in patients with cleft palate when compared to the controls, whereas no association was shown between rs642961, rs2236907, rs861019, and rs1044516 and non-syndromic orofacial clefts.

**Conclusion:**

The association found between rs2235371 and isolated cleft palate should be interpreted with caution due to the low number of individuals investigated, and more studies with larger sample size are needed to confirm these association. In addition, there is a lack of association of the rs642961, rs2236907 and rs861019 polymorphisms with non-syndromic orofacial clefts susceptibility.

## Resumo

**Introdução:**

As fendas orofaciais não sindrômicas possuem uma etiologia complexa devido à contribuição de fatores de risco genéticos e ambientais, assim como a interação entre eles. Dentre os mais de 15 loci de susceptibilidade para as fendas orofaciais não sindrômicas com considerável suporte estatístico e biológico, o IRF6 é o gene mais validado pela maioria dos estudos. Apesar disso, em populações geneticamente heterogêneas como a brasileira, a confirmação da associação entre as fendas orofaciais não sindrômicas e as variantes mais comuns do IRF6 ainda não é um fato consolidado e outras variantes não tão conhecidas IRF6 são pouco investigadas.

**Objetivo:**

O objetivo deste estudo foi investigar a associação de variados polimorfismos do IRF6 com o desenvolvimento das fendas orofaciais não sindrômicas em uma população do nordeste do Brasil.

**Método:**

Amostras de sangue de 186 pacientes com fendas orofaciais não sindrômicas e 182 controles do estado do Rio Grande do Norte, Brasil, foram obtidas para analisar os polimorfismos do IRF6 (rs2235371, rs642961, rs2236907, rs861019 e rs1044516) por reação em cadeia da polimerase em tempo real. Os pacientes com fendas orofaciais não sindrômicas foram classificados em fenda labiopalatina, fenda palatina isolada e fenda labial isolada.

**Resultados:**

As frequências genotípica e alélica do polimorfismo de único nucleotídeo rs2235371 no IRF6 mostraram-se significativamente diferentes em pacientes com fenda palatina isolada quando comparadas às dos controles, enquanto que nenhuma associação foi encontrada entre rs642961, rs2236907, rs861019 e rs1044516 e risco para o desenvolvimento das fendas orofaciais não sindrômicas.

**Conclusão:**

A associação encontrada entre rs2235371 e fenda palatina isolada deve ser interpretada com cautela devido ao baixo número de indivíduos investigados, sendo necessários mais estudos com um tamanho amostral maior para confirmar essa associação. Além disso, não foram encontradas associações significativas entre os demais polimorfismos do IRF6 rs642961, rs2236907, rs861019 e rs1044516 e a susceptibilidade às fendas orofaciais não sindrômicas.

## Introduction

Orofacial Clefts (OFCs) are the largest group of craniofacial malformations in humans and are a significant public health problem, due to the medical, psychological, social, and economic ramifications.^1^, ^2^, ^3^ OFCs include three general categories: those affecting the lip only (CL), those affecting the lip and palate (CLP), and those affecting the palate only (CP).^4^ However, previous genetic and embryological studies have suggested that CL and CLP, collectively known as CL/P, have distinct etiological mechanisms when compared to CP.^5^, ^6^ OFCs are characterized by incomplete formation or fusion of structures separating the nasal and oropharyngeal cavities and can affect the upper lip, the hard and/or soft palate, and other parts of the face.^7^

Most OFCs are not associated with additional malformations or anomalies and are thus classified as Non-Syndromic OFCs (NSOFCs). Despite the important influence of environmental risk factors, such as maternal consumption of alcohol and cigarettes, genetic factors have the biggest etiological contribution to NSOFC. Indeed, the first studies of Fogh-Anderson and then subsequent segregation analysis and twin studies have demonstrated a high rate of family occurrence, with a concordance rate of 40%–60% in monozygotic twins and 3%–5% in dizygotic twins.^8^, ^9^

To date, there has been considerable statistical and biological support for more than 15 NSOFC susceptibility loci. Of these, 1q32, which includes Interferon Regulatory Factor 6 (IRF6), was initially identified in candidate gene studies,^10^ and is the most validated locus in Genome-Wide Association Studies (GWAS) of NSOFC.^11^ Furthermore, structural mutations in IRF6 can also cause Van der Woude and popliteal pterygium syndromes.^12^ Analysis of animal models indicates that IRF6 plays an important role in facial development, participating in proliferation and differentiation of keratinocytes during the formation of the oral periderm and its spatio-temporal regulation is essential for appropriate palatal adhesion.^13^, ^14^

Several genetic polymorphisms of IRF6 have been associated with NSOFC risk in many populations, including Non-Syndromic Cleft Lip with or without cleft Palate (NSCL/P) subtype. The SNP, rs2235371, which changes valine to isoleucine at amino acid position 274 (V274I), was one of the first polymorphisms significantly associated with NSCL/P in Asian and Amerindian populations.^15^, ^16^ However, this result has not been replicated in other studies.^17^, ^18^, ^19^ The variant, rs642961, which is located within an enhancer ∼10 kb upstream of the IRF6 transcription start, was found to be a causative variant in people of European ancestry, being responsible for 18% of NSCL occurrence.^20^ The A allele of rs642961 was found to be significantly associated with NSCL/P in some studies,^21^, ^22^ but this has not been replicated in populations from Brazil, Iran, or Scandinavia.^19^, ^23^ Another GWAS has suggested evidence of an association between rs1044516 and NSCL/P in Asian populations, but not in other populations.^11^ This same polymorphism also showed evidence of an interaction with maternal tobacco smoke in Chinese case-parent trios.^24^

The association between IRF6 variants and NSOFC has been extensively explored in several populations in an effort to determine if IRF6 mutation screening should become a routine procedure, given its crucial role in OFCs. Nonetheless, in genetically heterogeneous populations, such as those in Brazil, the association between NSOFCs and common IRF6 variants has not been confirmed and unrecognized IRF6 variants are poorly investigated. Therefore, the aim of this study was to investigate the association of IRF6 polymorphisms (rs2235371, rs642961, rs2236907, rs861019 and rs1044516) with the development of NSOFC in a population from northeast Brazil. In addition, we evaluated the potential effect of allelic combinations of IRF6 polymorphisms on the development of NSOFC.

## Methods

### Subjects

The study population consisted of 186 NSOFC patients and 182 control individuals. NSOFC patients aged between 1 and 25 years of both genders were recruited from the Pediatric Endocrinology Unit, Onofre Lopes Hospital (HUOL) of the Federal University of Rio Grande do Norte (UFRN) and from the Varela Santiago Pediatric Hospital, both in Natal, Rio Grande do Norte, Brazil. The control group consisted of children above 5 years of age without a family history of clefts, recruited from public schools in Natal, Rio Grande do Norte, Brazil. NSOFC patients were classified as Cleft Lip and Palate (CLP), Cleft Palate only (CP), or Cleft Lip only (CL) groups according to the Fogh-Andersen scheme.^9^ A geneticist evaluated the patients and excluded those with characteristics of any associated syndrome.

The Ethics Committee on Human Research of the Federal University of Rio Grande do Norte approved the study under approval number 787.389. The study was performed according to the Declaration of Helsinki and resolution no. 66/12 of Brazil's National Health Council. Written informed consent was obtained from all adult subjects and parents or legal guardians of underaged patients and controls.

### Genetic analysis

Genomic DNA was isolated from peripheral blood leukocytes using a QIAamp DNA Blood Mini Kit (Qiagen, Valencia, CA, USA). Genotyping was performed by TaqMan® allelic discrimination using a 7500 Fast Real-Time PCR System (Applied Biosystems, Foster City, CA, USA). The pre-designed assays, C_15952140_10, C_2238941_10, C_11672849_20, and C_2500178_10 (Applied Biosystems, Foster City, CA, USA), were used to genotype the rs2235371, rs642961, rs2236907, and rs861019 polymorphisms of IRF6, respectively. For the rs1044516 polymorphism, primers and probes were designed using Primer Express® software (Applied Biosystems, Foster City, CA, USA). Primers were synthesized by Integrated DNA Technologies (Coralville, IA, USA) and probes by Applied Biosystems. The primers and probes for this assay are as follows: forward, 5′GAGGAATGGATGAGTGATTTGTGA3′; reverse, 5′ATGTACTCTCCCGGCATTCTGA3′; probe 1, 5′-FAM-TCTTAACTAGATACCCCGGTT-MGB-3′ and probe 2, 5′-VIC-TAGATAACCCGGTTTC-MGB-3′. To validate the genotyping method, 10% of the samples were randomly chosen for re-genotyping.

The polymorphisms genotyped in this study were chosen based on the following criteria: previously reported associations with OFCs, location within coding or putative regulatory regions, and known interactions with environmental factors. TagSNPs were used to identify common haplotypic variation within IRF6. rs2235371 is located in exon 7, rs642961 is located within an enhancer, rs2236907 is in an intronic region, rs861019 is within the 5′-UTR, and rs1044516 is within the 3′-UTR.

### Statistical analyses

Hardy-Weinberg equilibrium was evaluated using a chi-square test. Associations between polymorphisms and phenotype groups were assessed by calculating the Odds Ratio (OR) and its associated 95% CI using the SNPassoc package from the statistical software, R version 2.15.2 (R Foundation for Statistical Computing, Vienna, Austria). Pairwise Linkage Disequilibrium (LD) was computed as D′ for all SNPs using the Haploview program (version 4.2; Broad Institute, Cambridge, MA, USA). Haplotype block structure was determined using the confidence interval algorithm^25^ and haplotype frequencies were estimated using the expectation-maximization algorithm. A *p*-value <0.05 was considered significant.

## Results

Table 1 shows the clinical characteristics of the case and control groups. Among the 186 NSOFC patients, there was a predominance of males, notably with CL and CLP types and an excess of females with the CP type, which is in accordance with previous studies. There were no differences in age distribution between the case and control groups. CLP was the cleft type most reported, followed by CP and CL. Approximately one third of the patients had a family history of clefts.

**Table 1:** Clinical characteristics of studied groups.

	Case (*n* = 186)	Control (*n* = 182)	*p*-value
*Gender*^a^			
Male	119 (64.1%)	90 (49.4%)	<0.001
Female	67 (35.9%)	92 (50.6%)	

*Age*^b^	8.1 ± 7.5	11.7 ± 6.2	0.175

*Cleft types*			
Cleft lip and palate (CLP)	106 (57.0%)		
Male	84 (79.2%)		
Female	22 (20.7%)		
Cleft Lip only (CL)	42 (22.6%)		
Male	28 (66.6%)		
Female	14 (33.3%)		
Cleft Palate only (CP)	38 (20.4%)		
Male	06 (15.7%)		
Female	32 (84.2%)		

a Chi-square test.

b *t*-test.

All studied polymorphisms were in Hardy-Weinberg equilibrium in both groups. Allelic and genotypic distributions of the IRF6 polymorphisms (rs2235371, rs642961, rs2236907, rs861019, and rs1044516) in cases and controls are presented in Table 2. Taking into account embryological differences of NSOFC, the frequencies of IRF6 polymorphisms in the case group were analyzed in CL/P (CL + CLP) and CP subgroups. There was a significant association between rs2235371 and CP, with The T allele (*p* = 0.004) and TT genotype (*p* = 0.016) being significantly more frequent in the CP group than the control group.

**Table 2:** Genotypic frequencies of IRF6 polymorphisms in studied groups.

Polymorphims	Genotypes	Control	CLP + CL (%)	OR (95% CI)	*p*-value	CP (%)	OR (95% CI)	*p*-value
rs2235371	CC	163 (89.6%)	129 (87.1%)	1.00	0.247	29 (76.3%)	1.00	0.016^a^
	CT	17 (9.3%)	16 (10.8%)	1.61 (0.84–3.10)		05 (13.2%)	1.65 (0.57–4.83)	
	TT	02 (1.1%)	03 (2.0%)	2.19 (0.39–12.12)		04 (10.5%)	11.24 (1.97–64.22)	
Minor allele carrier (CT + TT)		19 (10.4%)	19 (12.8%)	1.22 (0.54–1.72)	0.887	09 (23.7%)	2.35 (0.23–3.71)	0.033^a^
Minor allele frequency		21 (5.75%)	22 (7.4%)	1.16 (0.69–1.55)	0.762	13 (17.1%)	3.01 (0.97–8.97)	0.004^a^
rs642961	GG	133 (73.5%)	112 (74.3%)	1.00	0.754	30 (76.9%)	1.00	0.590
	GA	43 (23.8%)	29 (19.5%)	0.84 (0.51–1.39)		07 (17.9%)	0.72 (0.30–1.76)	
	AA	05 (2.8%)	06 (6.2%)	1.17 (0.35–3.94)		02 (5.1%)	1.77 (0.33–9.58)	
Minor allele carrier (GA + AA)		48 (26.6%)	35 (25.7%)	1.56 (0.98–5.67)	0.587	09 (23.0%)	2.34 (0.96–5.98)	0.672
Minor allele frequency		53 (14.7%)	42 (15.9%)	1.89 (0.34–3.27)	0.892	11 (14.0%)	3.57 (0.64–4.57)	0.748
rs2236907	CC	93 (51.3%)	65 (43.6%)	1.00	0.216	20 (51.3%)	1.00	0.384
	CA	57 (31.7%)	51 (34.8%)	0.80 (0.50–1.26)		10 (25.6%)	1.24 (0.54–2.84)	
	AA	28 (15.5%)	32 (21.6%)	1.31 (0.71–2.42)		09 (23.1%)	2.04 (0.74–5.62)	
Minor allele carrier (CA + AA)		85 (47.2%)	83 (56.4%)	2.01 (1.17–3.12)	0.465	19 (48.7%)	3.25 (0.12–12.89)	0.872
Minor allele frequency		113 (31.3%)	115 (39.0%)	1.69 (0.82–2.89)	0.567	28 (35.9%)	2.12 (0.45–5.12)	0.723
rs861019	AA	85 (46.1%)	72 (48.9%)	1.00	0.827	20 (52.6%)	1.00	0.396
	AG	64 (35.0%)	50 (34.2%)	1.08 (0.69–1.72)		15 (39.4%)	1.01 (0.48–2.13)	
	GG	33 (18.9%)	26 (16.9%)	0.91 (0.50–1.66)		03 (7.8%)	0.49 (0.15–1.61)	
Minor allele carrier (CA + AA)		97 (53.9%)	76 (51.1%)	1.98 (1.21–2.93)	0.657	18 (47.2%)	2.78 (1.12–3.27)	0.827
Minor allele frequency		130 (36.4%)	106 (34.0%)	1.23 (0.67–3.56)	0.734	23 (27.5%)	3.89 (0.45–6.34)	0.645
rs1044516	AA	99 (55.0%)	88 (59.4%)	1.00	0.645	20 (51.3%)	1.00	0.893
	AC	71 (39.4%)	52 (35.1%)	0.82 (0.53–1.26)		17 (43.6%)	1.19 (0.58–2.42)	
	CC	10 (5.6%)	08 (5.4%)	0.84 (0.33–2.15)		02 (5.1%)	0.99 (0.20–4.87)	
Minor allele carrier (AG + GG)	81 (45.0%)	60 (40.5%)	2.10 (1.02–3.65)	0.362	19 (48.7%)	1.87 (1.65–3.01)	0.574	
Minor allele frequency	91 (25.3%)	68 (22.9%)	3.21 (0.75–4.96)	0.824	21 (26.9%)	3.15 (0.89–4.56)	0.897	

a Significant association, *p* < 0.05. CLP, Cleft Lip and Palate; CL, Cleft Lip only; CP, Cleft Palate only.

**Figure 1:**
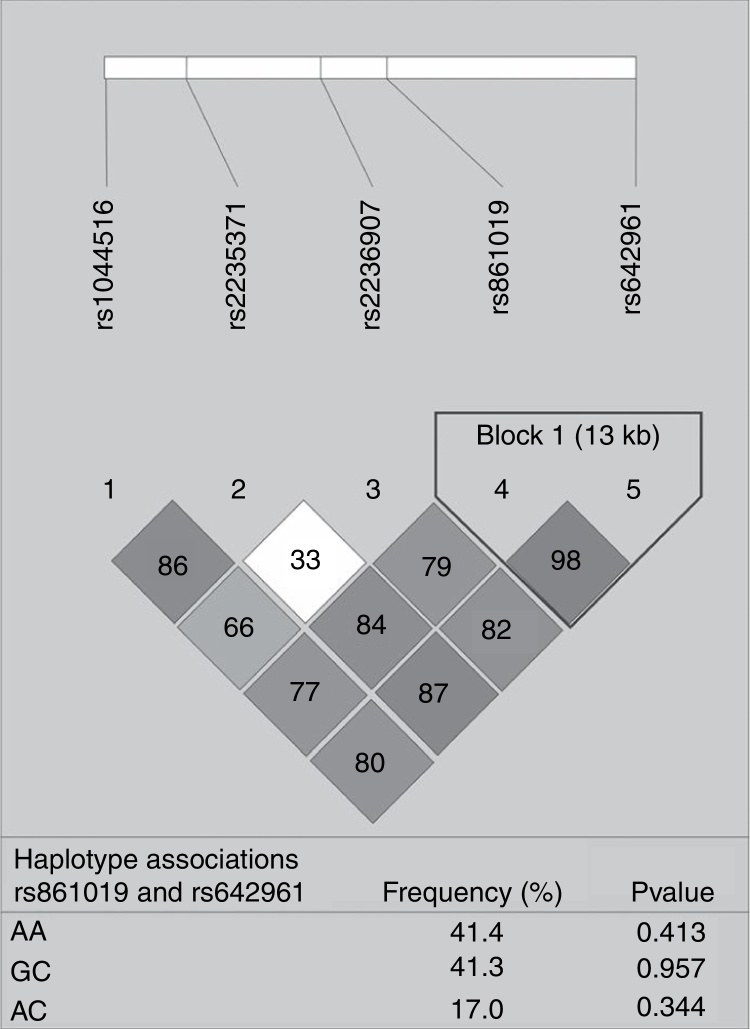
Pairwise linkage disequilibrium plot of IRF6/rs1044516, rs2235371, rs2236907, rs861019, and rs642961 in the combined sample (cases & controls) showing *r*^2^ (×100) values. The block is designed according to the internally developed solid spine of linkage disequilibrium (LD). The value within each diamond represents the pairwise correlation between pairs of Single-nucleotide polymorphisms (SNPs) (measured as 100 × *r*^2^) defined by the upper left and the upper right sides of the diamond. Below are showed the frequency of each haplotype.

None of the polymorphisms studied showed differences in allele or genotype frequencies between the total case group and controls (data not shown). The LD analysis of IRF6 polymorphisms is shown in Fig. 1. One haplotype block was constructed between rs861019 and rs642961; however, it did not show a significant association with NSOFC (*p* > 0.05).

## Discussion

NSOFCs have been studied for many years in an attempt to understand their etiology since they constitute the majority of OFC cases. Genetic factors are known to play an important role in the development of OFCs, given the increased occurrence risk in relatives up to the third degree, when compared to the general population.^26^ In previous studies, variation at IRF6 has been responsible for a considerable percentage of the genetic contribution to NSOFCs. IRF6 variants have also been associated with the most severe sub-phenotypes and symmetrical shape variations in these patients, including a more protrusive forehead, a shorter nose, and a steep mandibular plane.^10^, ^22^, ^27^, ^28^, ^29^, ^30^ However, studies investigating IRF6 polymorphisms in different patient populations have given divergent results.^17^, ^31^ These findings are especially discordant in studies with mixed populations, such as those in Brazil, where results have varied according to the geographic region and ethnicity studied.^16^, ^19^, ^32^ Thus, in order to better understand the association of IRF6 with NSOFC, we evaluated the rs2235371, rs642961, rs2236907, rs861019 and rs1044516 polymorphisms in a Brazilian population.

None of the alleles or genotypes at the IRF6 polymorphisms evaluated here were associated with an increased risk of NSOFC in the studied population. Although an association was found between rs2235371 and the CP group, this result should be interpreted with caution, since the small number of individuals in this group affected the reliability of the result, and the association lost significance after correction for multiple tests. A similar result for rs2235371 in CP patients has been reported in an Indian population.^33^

Several studies have reported associations between rs2235371 and NSOFC risk, especially in Asian populations. A GWAS performed by Sun et al.^15^ showed that the rs2235371 variant was consistently associated with NSCL/P and it appeared to have a higher importance than the previously identified causal variant, rs642961, in Chinese populations.^15^ The A allele and AA and AG genotypes at rs2235371 were associated with a decreased risk of NSCL/P in this population when compared to GG genotypes. However, in Caucasians, these genotypes are not significantly associated with NSOFCs.^31^ The low frequency of the allele coding for isoleucine in populations of European descent is a possible explanation for these divergent results among populations.^20^ Indeed, the rs2235371 seems not to be causal, but rather in LD with another causal variant in IRF6.^27^

Among studies performed in Brazilian populations, Letra et al.^32^ investigated 142 case-parent trios from Sao Paulo and found a positive association between the rs2235371 polymorphism and the specific sub-phenotypes, complete left CLP and CP cases with impaction of permanent teeth. Although IRF6 was not a strong risk factor in that study, the authors suggested that IRF6 variants contributed to the specific sub-phenotype by controlling the affected side of unilateral cleft development. De Souza et al.^16^ studied 673 individuals from southern, southeastern, and northeastern Brazil and observed a significant association between the G allele at rs2235371 and CL/P and CL, but not CP in subjects with European ancestry. In contrast, Paranaiba et al.^19^ studied 228 patients from Minas Gerais, Brazil and observed no association between rs2235371 and NSOFCs.

The rs642961 SNP has been shown to be associated with NSCL/P in both European and Asian populations.^21^, ^31^ The A allele and AA and GA genotypes at rs642961 were found to be significantly associated with NSCL/P, especially in CL individuals.^20^, ^31^ Do Rego-Borges et al.^34^ studied 293 unrelated NSCL/P patients from a Brazilian population with a high level of African ancestry (Bahia State) and found a marginal association with rs642961 heterozygotes (GA), which they attributed to the signiﬁcant European-derived ancestry component in this population.

However, as in the present study, many investigations have failed to replicate this association with rs642961. In the present study, the lack of association may be attributed to the smaller sample size and the effect of clinical heterogeneity among affected individuals (e.g., variation in disease severity).^22^ In fact, some studies only found an association with this SNP when analyzing specific cleft subtypes or when analyzing haplotypic combinations with other polymorphisms. Sun et al.^15^ demonstrated that the effect of rs642961 can be weakened by rs2235371, suggesting that the key variants may be located at rs2235371 in related haplotypes.

Brito et al.^35^ studied 563 NSCL/P patients from ﬁve different Brazilian cities and found no signiﬁcant allelic association with rs642961. However, when stratiﬁed by sub-phenotypes, an association was found between rs642961 and CL was observed. De Souza et al.^16^ did not report an association when rs642961 was analyzed individually, but haplotype analyses showed an association of the A allele of rs642961 and the G allele of rs2235371 with an increased risk of NSCL/P in children and their mothers.

Although it is known that the haplotype information can increase the resolution of genetic data compared with examining SNPs individually, in our study there was no significant association with the constructed haplotype and NSOFC risk.

## Conclusions

Although contributions from single genes, such as IRF6, now seem to explain approximately 15% of NSOFC cases, in the present case-control study, there were no significant associations between rs2235371, rs642961, rs2236907, rs861019, or rs1044516 SNPs in IRF6 and NSOFCs. Despite finding an association between rs2235371 and CP risk in our patients, additional studies in similar populations, but with larger sample sizes, are required to further investigate potential associations with different NSOFC sub-phenotypes and to identify the pattern of IRF6 genetic variants in admixed populations, such as those seen in Brazil.

## Funding

This work was supported by the 10.13039/501100003593National Council for Scientific and Technological Development CNPq), Brazil, grant number 477608/2011-6.

## Conflicts of interest

The authors declare no conflicts of interest.
